# Population Pharmacokinetics of Vancomycin and Meropenem in Pediatric Extracorporeal Membrane Oxygenation Support

**DOI:** 10.3389/fphar.2021.709332

**Published:** 2021-08-13

**Authors:** Brenda Zylbersztajn, Suzanne Parker, Daniel Navea, Giannina Izquierdo, Paula Ortiz, Juan Pablo Torres, Cristian Fajardo, Rodrigo Diaz, Cristian Valverde, Jason Roberts

**Affiliations:** ^1^ Pediatric Intensive Care Unit, Clinica Las Condes, Santiago, Chile; ^2^ UQ Centre for Clinical Research, The University of Queensland, Brisbane, QLD, Australia; ^3^ Laboratory, Clinica Las Condes, Santiago, Chile; ^4^ Department of Infectious Disease, Clinica Las Condes, Santiago, Chile; ^5^ Pediatric Intensive Care Unit, Roberto Del Rio Hospital, SantiagoChile; ^6^ Intensive Care Unit, Clinica Las Condes, Santiago, Chile; ^7^ Department of Intensive Care Medicine, Royal Brisbane & Women’s Hospital, Brisbane, QLD, Australia; ^8^ Division of Anaesthesiology Critical Care Emergency and Pain Medicine, Nîmes University Hospital, University of Montpellier, Nîmes, France; ^9^ Department of Pharmacy, Royal Brisbane & Women’s Hospital, Brisbane, QLD, Australia

**Keywords:** vancomycin, meropenem, pediatric, extracorporeal membrane oxygenation, pharmacokinetic, pharmacodynamic

## Abstract

**Objective:** Describe primary pharmacokinetic/pharmacodynamic (PK/PD) parameters of vancomycin and meropenem in pediatric patients undergoing ECMO and analyze utilized dosing to reach PK/PD target.

**Design:** Prospective, multicentric, population PK analysis.

**Setting:** Two hospitals with pediatric intensive care unit.

**Patients:** Pediatric patients (1 month - 15 years old) receiving vancomycin and meropenem for empiric or definitive infection treatment while ECMO support.

**Measurements and Main Results:** Four serum concentration were obtained for patients receiving vancomycin (*n* = 9) and three for meropenem (*n* = 9). The PK/PD target for vancomycin was a ratio of the area under the curve to the minimal inhibitory concentration (AUC/MIC) of >400, and for meropenem was 4 times above MIC for 50% of the dosing interval (*f*T_50%_ > 4xMIC). Pharmacokinetic modeling was performed using PMetrics 1.5.0. We included nine patients, with 11 PK profiles for each antimicrobial. The median age of patients was 4 years old (2 months - 13 years) and 45% were male. Creatinine clearance (CL) was 183 (30–550) ml/min/1.73 m^2^. The median dose was 13.6 (range 10–15) mg/kg every 6–12 h and 40 mg/kg every 8–12 h for vancomycin and meropenem, respectively. Two compartment models were fitted. Weight was included as a covariate on volume of the central compartment (Vc) for meropenem. Weight was included as a covariate on both Vc and clearance (CL) and serum creatinine was also included as a covariate on CL for vancomycin. The pharmacokinetic parameters CL and Vc were 0.139 ± 0.102 L/h/kg and 0.289 ± 0.295 L/kg for meropenem and 0.060 ± 0.055 L/h/kg and 0.419 ± 0.280 L/kg for vancomycin, respectively. Across each dosing interval 91% of patients achieved the PK/PD targets for adequate exposure for meropenem and 63.6% for vancomycin.

**Conclusion:** Pharmacokinetic/pharmacodynamic objectives for vancomycin were achieved partially with conventional doses and higher dosing with extended infusion were needed in the case of meropenem.

## Introduction

Extracorporeal membrane oxygenation (ECMO) allows respiratory or cardiac support for critically ill patients (Fraser et al., 2012). ECMO is not considered a treatment cure, although it sustains life while the underlying pathology is being managed. It also provides a bridge to heart or lung transplantation or full organ recovery (Abdul-Aziz et al., 2019).

Patients on ECMO are critically ill and receive multiple drugs; therefore, understanding the pharmacokinetic (PK) alterations that occur on ECMO is crucial. Different anti-infective drugs are frequently used in patients on ECMO because multiple intravenous devices and long intensive care unit (ICU) stay (Sherwin et al., 2016).

Vancomycin is a frequently used first-line antibiotic for treating infections caused by methicillin-resistant *Staphylococcus aureus* or other Gram-positive suceptible bacteria (Rybak et al., 2009). The pharmacokinetic/pharmacodynamic (PK/PD) target that best predicts the efficacy of vancomycin is the ratio of the area under the curve to the minimal inhibitory concentration (AUC/MIC) (Liu et al., 2011). Previous vancomycin PK reported data in newborn on ECMO have shown an increased volume of distribution (Vd) and decreased clearance (CL) (Buck, 1998), but conclusions could not be extrapolated to pediatric patients because organ development and physiological factors affects drug’s PK in newborns (Lu and Rosenbaum, 2014).

Meropenem is a carbapenem antibiotic widely used as first-line therapy for infections caused by nosocomial Gram-negative bacilli such as extended-spectrum β-lactamase (ESBL) - producing Enterobacteriaceae. Meropenem is a time-dependent antibiotic and there PK/PD target is the time that free drug concentrations remain above the minimum inhibitory concentration (MIC) at the site of infection (*f*T % >MIC). To ensure a bactericidal effect, free drug concentration should be about 4 to 6 times the MIC, at least 40% of the dosing interval (Cies et al., 2015). Recent data showed that prolonged or continuous infusion of meropenem can achieve a higher *f*T > MIC and might be associated with improved microbiological outcomes (Drusano, 2003; Tam et al., 2003; Drusano, 2004; Lodise et al., 2006; Cies et al., 2015; Kongthavonsakul et al., 2016; Rapp et al., 2020).

Most of the meropenem PK data from children are limited to healthy volunteers or non ICU patients. The available pharmacokinetic data for pediatric ICU patients demonstrates greater CL and Vd (Cies et al., 2017). There are scarce data describing the impact of ECMO on the pharmacokinetics and dosing requirements of meropenem in critically ill pediatric patients, and most of this data is derived from case reports (Cies et al., 2014; Cies et al., 2016; Saito et al., 2020).

Currently, there are no guidelines on antimicrobial dosing for pediatric patients undergoing ECMO. Dosing regimens are based on recommendations for critically ill pediatric patients, data from adults or newborns and considerations of physicochemical characteristics, plasma concentrations of different drugs and clinical outcomes (Zylbersztajn et al., 2018). The aim of this study was to describe the population pharmacokinetics of vancomycin and meropenem in pediatric patients undergoing ECMO, as well as the achievement of therapeutic exposures with currently used dosing regimens.

## Materials and Methods

This study was a prospective, multicentric, PK/PD study. It included pediatric patients who were admitted to the pediatric ICU between July 2017 and November 2019 at two hospitals in Santiago, Chile. The Ethical Committee for Research of Clinica Las Condes approved this prospective study (approval number: M012017) and required signed written informed consent from each patient’s parents. Patients younger than 15 years of age requiring ECMO and antimicrobial treatment with vancomycin and or meropenem for suspected or confirmed infection were included. Neonates were excluded.

Meropenem was administered as a 3-h infusion with intravenous doses of 20–40 mg/kg every 8 or 12 h. Vancomycin was administered as a 2-h infusion at 10–15 mg/kg intravenously every 6 or 12 h. Both antibiotics’ doses were adjusted according to renal function. Blood samples were collected at 1 and 3 h post-infusion and before the following dose for meropenem. For vancomycin, blood samples were collected at 0.5, 1, and 2 h post-infusion and before the following dose. Samples were collected under steady state conditions: 24 h for meropenem and 48 h for vancomycin from the beginning of the treatment.

Unbound vancomycin plasma concentration were determined by an immunoenzymatic assay (Cobas c 311, Roche, Japan) with a precision of 2.4%, accuracy of 1.8% and lower limit of quantification (LLOQD) of 1.7 mcg/mL. Unbound meropenem plasma concentrations were determined by high-performance liquid chromatography (Agilent Infinity 1,260, Santa Clara, United States) with a UV detector. The methodology was adapted to the equipment conditions from available publications (Elkhaïli et al., 1996; McWhinney et al., 2010; Wolff et al., 2013) and validated according to the recommendations of the Bioanalytical Method Validation Guidance for Industry (U.S. Department of Health and Human Services, 2018). The stationary phase was a Zorbax Eclipse Plus C18 column (3.5 μm, 75 × 4.6 mm) and the mobile phase was acetonitrile with 10 mM ammonium acetate (pH 4.0) (5:95, organic solvent:buffer initiallity) in gradient to minute 7(50:50, organic solvent:buffer). Meropenem was detected by UV detection at 298 nm. The flow rate was 1.0 ml/min. The retention time of meropenem was 3.5 min. Standard curves were prepared in blank human plasma. The standard curve was linear between 0.2 ug/mL and 100 ug/mL. Meropenem in plasma samples was quantified using the peak area of standard samples for calibrations. The precision of the methodology was 0.6% and accuracy was 0.5%. The lower limit of quantification (LLOQD) for meropenem was determined by replicate of 10 matrix blank samples, determining the standard deviation (SD) of the response obtained, the result was expressed as 10 times the SD obtained, reported as 0.16 ug/mL.

For both analytes, the measurement was performed on the free-drug, non-protein bound fraction. In the case of vancomycin through a specific colorimetric reaction and meropenem through the extraction of the free-drug fraction by organic solvent and subsequent purification of the sample.

### Model Development

Pharmacokinetic modelling was performed using PMetrics 1.5.0 with RStudio 0.99.902 and digital compiler Gfortran 5.2. We considered 33 plasma samples for meropenem and 44 plasma samples for vancomycin. For the population pharmacokinetic analysis, one-, two- and three-compartment models were fitted using plasma meropenem and vancomycin concentration data, using non-parametric adaptive grid subroutines from the PMetrics package for R (Los Angeles CA, United States). Primary pharmacokinetic parameters of CL and Vd for the central compartment were calculated. Elimination from the central compartment and inter-compartmental distribution were modelled as first-order processes. Inter-compartmental distribution was described as rate of transfer from the unbound compartment to a peripheral compartment (Kcp) and rate of transfer from a peripheral compartment to the unbound compartment (Kpc). Additive (lambda) and multiplicative (gamma) error models were tested using a polynomial equation for standard deviation as a function of observed concentration, Y. (SD = C_0_ + C_1_.Y), with observation weighting performed as error = SD. gamma or error = (SD^2^+lambda^2^)^0.5^. Volume of the peripheral compartment (Vp) was calculated as (Vc*Kcp)/Kpc, where Vc is the volume of the central compartment. Total Vd was calculated as the sum of Vc and Vp.

### Model Diagnostics

We selected the final model on the basis of minimizing the Akaike Information Criterion (AIC) and likelihood of the nested models (-2*Log-likelihood, -2*LL), with both criteria penalized by the number of parameters in the model. A reduction of -2*LL as calculated within Pmetrics was used for statistical comparison of nested models. We also factored bias (mean weighted predicted-observed error) and imprecision (bias-adjusted, mean weighted squared predicted-observed error) and correlation co-efficient into the selection of the final model. Shrinkage was calculated as the percentage of total variance in each model probability distribution.

### Covariate Screening

Covariate model building was performed using sequential assessment of biologically plausible clinical characteristics. Continuous data was used for all covariates tested, with the exception of gender, which was male/female. The association of the covariates versus parameters was assessed by regression with linear or non-linear associations able to be used in the covariate model testing with inclusion based upon biological plausibility and a statistically significant improvement in log-likelihood. Inclusion age (months), weight (kg), height (cm), gender, C-reactive protein (mg/L), albumin (g/L), total bilirubin (mg/dl), serum creatinine (mg/dl), 24-h hydric balance (ml).

For the PK/PD target attainment assessment, AUC of vancomycin was calculated using Pmetrics. For meropenem analysis, we used the plasma concentration at 1 h after the end of a 3-h infusion of the drug corresponding to a dosing interval of 50%. The target exposure for vancomycin was an AUC/MIC ratio >400 using the MIC of *Staphylococcus aureus* of 1 mcg/mL, and that for meropenem was plasma concentrations > 4xMIC for at least 50% of the dosing interval, using the MIC of *Pseudomonas aeruginosa* of 2 mcg/mL.

## Results

**Nine** patients were included in the pharmacokinetic modelling. The median age was 4 years old (2 months - 13 years) and 45% of patients were male. The median estimated creatinine clearance was 178 (30–239) ml/min/1.73 m^2^. The median doses was 54 (20–60) mg/kg/day in 2–4 divided doses for vancomycin and 120 (60–120) mg/kg/day in 2-3 divided doses for meropenem. Two patients received only vancomycin and two patients received only meropenem. Six patients completed one treatment of meropenem and vancomycin. One patient received meropenem and vancomycin in three different occasions. We performed a total of 11 PK profiles for each antimicrobial. Demographic and clinical characteristics of patients are reported in Tables 1 and 2.

**TABLE 1:** Patient characteristics.

	Vancomycin	Meropenem
Female (n)	6	5
Male (n)	3	4
ECMO PK profiles (n)	11	11
ECMO VA (n)	5	5
ECMO VV (n)	4	4
Oxygenator	polymethylpentene, phosphorylcholine coating	
CRRT (n)	2	4
Empiric treatment	8	6
Pneumonia	7	6
Endocarditis	1	1
Refractory septic shock	1	2

Number (n); extracorporeal membrane oxygenation (ECMO); pharmacokinetic (PK); veno-venous (VV); veno-arterial (VA); continuous renal replacement therapy (CRRT).

**TABLE 2:** Patient characteristics.

	Vancomycin			Meropenem		
Characteristic	Mean	Median	Range	Mean	Median	Range
Age (months)	50.4	24	2–132	68.3	48	2–165
Weight (kg)	15.5	10	3.5–37	20.4	16	3.5–45
Height (cm)	92.7	82	50–140	103	100	50–155
Serum creatinine (mg/dl)	0.34	0.22	0.08–1.23	0.41	0.3	0.08–1.27
Serum albumin (g/L)	3.8	3.55	2.8–5.4	3.6	3.5	2.6–5.4
Total bilirubin (mg/dl)	3.5	0.86	0.31–30.1	3.7	0.95	0.41–30.1
C-reactive protein (mg/L)	60.1	47	1.2–248	90.4	57.5	1.2–423
24 h hydric fluid balance (ml)	56.4	37.5	-1,203 – 1,220	-79.2	4.5	-2,615 – 1,170

**FIGURE 1:**
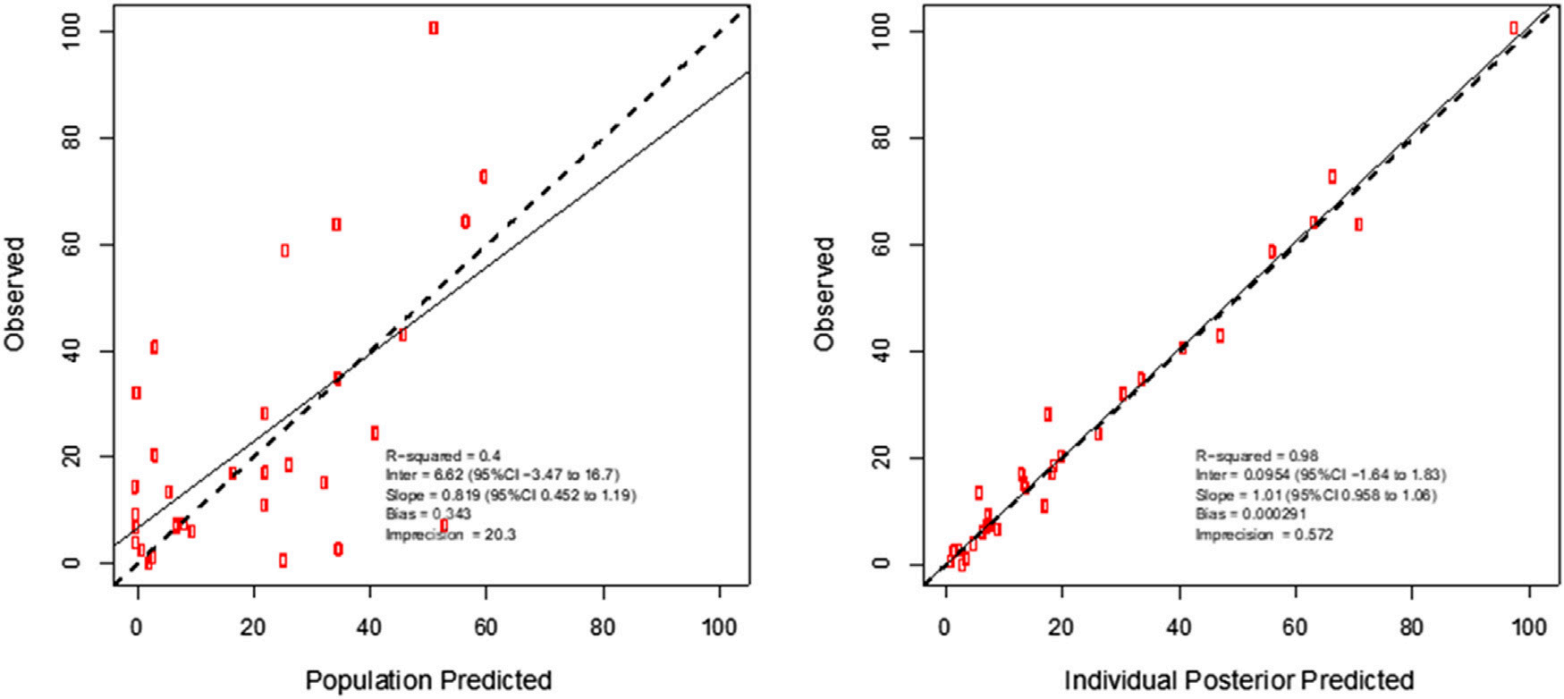
Individual predicted concentration diagnostic plots for the final covariate model for plasma meropenem concentrations. Data are presented in mg/L.

**FIGURE 2:**
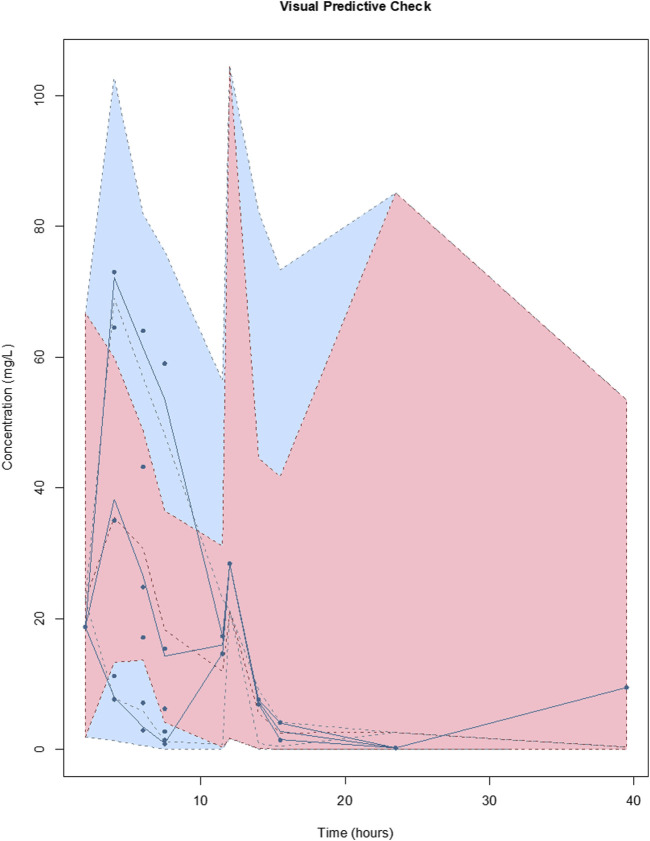
Visual predictive checks of the final covariate model for meropenem. The lines represent the percentiles of 1,000 simulated meropenem concentration-time profiles superimposed with observed meropenem concentrations (circles). The blue shading around the lines represents the 95% CI around each percentile. The distribution of the simulated concentration profiles is similar to that of the observed concentrations, with all of observed concentrations between 5th and 95th simulated percentiles, respectively, suggesting that the model describes the data adequately.

For meropenem the inclusion of a second compartment was accepted on the basis of a decrease in the log-likelihood of 10.1. The inclusion of total body weight as a covariate on central volume (Vw; *r* ^2^ = 0.17) was accepted on the basis of a decrease in log-likelihood of 44.6, as well as improvements to the slope and bias on the observed vs predicted plots. The diagnostic plots to confirm the goodness-of-fit of the final covariate model were considered acceptable and are shown in Figure 1. The visual predictive check plot is provided in Figure 2. The final model is reported in Table 3.

**TABLE 3:** Pmetrics model for the final covariate model for meropenem.

#Primary variables
CL,0.1.7
V,2,3.5
KCP,0.1.2.8
KPC,0,2.8
#Covariates
WGT
#Secondary variables
Vw = V*(WGT/20.4)
Ke = CL/Vw
#Output equations
Y (1) = X (1)/Vw
#Error model
G = 1
0.2.0.1,0,0

CL, clearance from the central compartment; V, volume of the central compartment (L); Kcp, first-order rate constant for distribution from central to peripheral compartment (h^−1^); Kpc, first-order rate constant for distribution from peripheral to central compartment (h^−1^); WGT, total body weight; Vw, typical estimate of volume of the central compartment for a total body weight of 20.4 kg; Ke, first-order elimination rate constant (h^−1^); Y (1), concentration of drug in the central compartment; Error, each observation is weighted by 1/(Error)^2^ using a multiplicative error model (Error = SD*gamma), where SD is the standard deviation of each observation which is modelled by a polynomial equation with coefficients of the assay error specified in the bottom rows for unbound meropenem concentrations and G (gamma) is a value relating to extra process noise related to the observation, such as mis-specified dosing and observation times.

**FIGURE 3:**
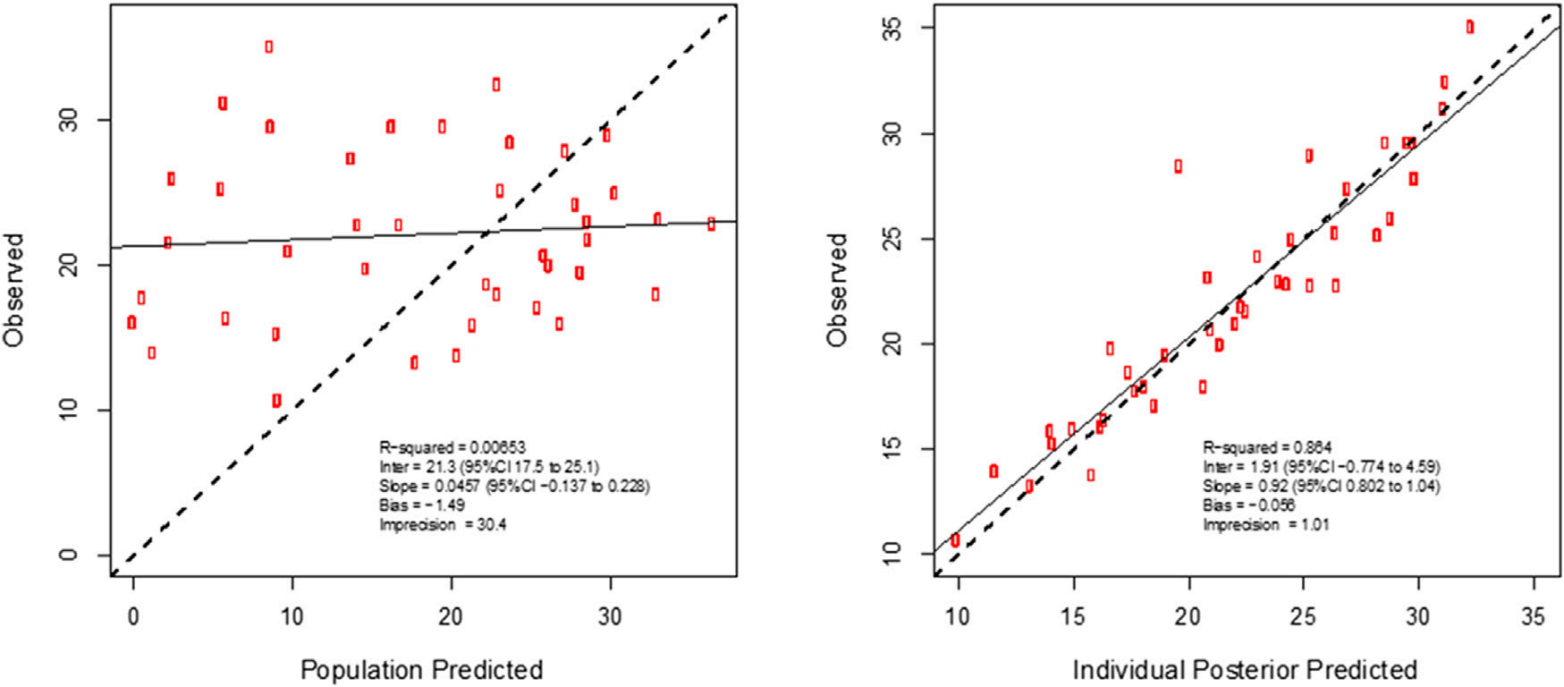
Individual predicted concentration diagnostic plots for the final covariate model for plasma vancomycin concentrations. Data are presented in mg/L.

**FIGURE 4:**
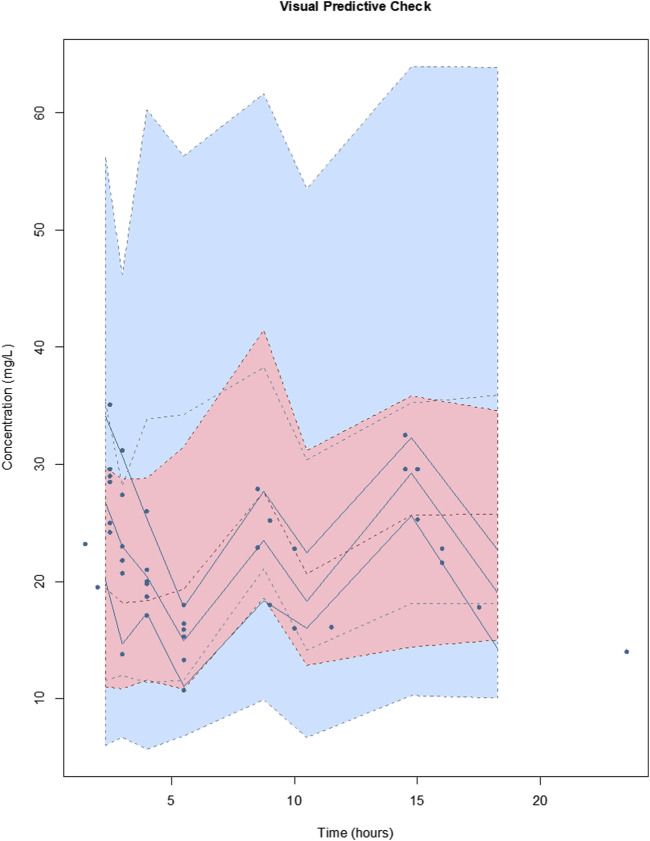
Visual predictive checks of the final covariate model for vancomycin. The lines represent the percentiles of 1,000 simulated vancomycin concentration-time profiles superimposed with observed vancomycin concentrations (circles). The blue shading around the lines represents the 95% CI around each percentile. The distribution of the simulated concentration profiles is similar to that of the observed concentrations, with all of observed concentrations between 5th and 95th simulated percentiles, respectively, suggesting that the model describes the data adequately.

For vancomycin the inclusion of a second compartment was accepted on the basis of a decrease in log-likelihood of 31.0. The inclusion of total body weight as a covariate on central volume (Vw; *r* ^2^ = 0.71) was accepted on the basis of a decrease in log-likelihood of 21.3. The inclusion of total body weight and serum creatinine as covariates on clearance (CLw; *r* ^2^ = 0.71 and 0.29, respectively) was accepted on the basis of a decrease in log-likelihood of 78.9 and 17.9. The diagnostic plots to confirm the goodness-of-fit of the final covariate model were considered acceptable and are shown in Figure 3. The visual predictive check plot is provided in Figure 4. The final model is reported in Table 4.

**TABLE 4:** Pmetrics model for the final covariate model for vancomycin.

#Primary variables
CL,0.3.0.7
V,2.4.6
Kcp,0.12.1
Kpc,0.01.0.7
#Covariates
WGT
CREA
#Secondary variables
CLw = CL*(wgt/15.5)*(0.56*(0.34/crea))
Vw = V*(wgt/15.5)
Ke = CLw/Vw
#Output equations
Y (1) = X (1)/Vw
#Error model
G = 3
2,1,0,0

CL, clearance from the central compartment; V, volume of the central compartment (L); Kcp, first-order rate constant for distribution from central to peripheral compartment (h^−1^); Kpc, first-order rate constant for distribution from peripheral to central compartment (h^−1^); WGT, total body weight; CREA, serum creatine concentration (mg/dl); CLw, typical estimate of clearance from the central compartment for a total body weight of 15.5 kg and serum creatinine of 0.34 mg/dl; Vw, typical estimate of volume of the central compartment for a total body weight of 15.5 kg; Ke, first-order elimination rate constant (h^−1^); Y (1), concentration of drug in the central compartment; Error, each observation is weighted by 1/(Error)^2^ using a multiplicative error model (Error = SD*gamma), where SD is the standard deviation of each observation which is modelled by a polynomial equation with coefficients of the assay error specified in the bottom rows for vancomycin concentrations and G (gamma) is a value relating to extra process noise related to the observation, such as mis-specified dosing and observation times.

There were no plasma concentration below the LLOQD for either antimicrobial. The primary pharmacokinetic parameters are summarized in Table 5 for meropenem and Table 6 for vancomycin.

**TABLE 5:** Population pharmacokinetic model data of meropenem concentrations of paediatric patients undergoing extracorporeal membrane oxygenation.

PK parameter	Units	Mean	SD	Median	CV%	Shrink%
CL	L.h^−1^. kg^−1^	0.139	0.102	0.144	73.3	2.2
Vc	L. kg^−1^	0.289	0.295	0.289	72.0	4.8
Kcp	h^−1^	1.37	0.992	1.30	72.6	25.2
Kpc	h^−1^	1.25	0.984	1.34	79.0	20.6
Vp (Vc*kcp/kpc)	L. kg^−1^	0.317	0.297			
Vd (vc + vp)	L.kg^−1^	0.606	0.592			

Pharmacokinetic (PK); clearance of meropenem (CL); central volume of distribution of meropenem (Vc); rate of transfer from the central compartment to a peripheral compartment (Kcp); rate of transfer from a peripheral compartment to the central compartment (Kpc); volume of distribution of meropenem (Vd); standard deviation (SD); coefficient of variation (CV%); model shrinkage (Shrink%).

**TABLE 6:** Population pharmacokinetic model data of vancomycin concentrations of paediatric patients undergoing extracorporeal membrane oxygenation.

PK parameter	Units	Mean	SD	Median	CV%	Shrink%
CL	L.h^−1^.kg^−1^	0.060	0.055	0.061	92.2	8.1
Vc	L.kg^−1^	0.419	0.280	0.397	61.0	8.7
Kcp	h^−1^	0.483	0.304	0.481	63.0	7.0
Kpc	h^−1^	0.248	0.114	0.286	45.8	13.9
Vp (Vc*kcp/kpc)	L.h^−1^	0.816	0.747			
Vd (vc + vp)	L.kg^−1^	1.235	1.027			

Pharmacokinetic (PK); clearance of vancomycin (CL); central volume of distribution of vancomycin (Vc); rate of transfer from the central compartment to a peripheral compartment (Kcp); rate of transfer from a peripheral compartment to the central compartment (Kpc); volume of distribution of vancomycin (Vd); standard deviation (SD); model shrinkage (Shrink%).

For meropenem, 91% of PK profiles achieved therapeutic exposures based on the PK/PD targets of *f*T _50%_ > 4 x MIC and for vancomycin only 63.6% of PK profiles reached AUC/MIC>400. The PK/PD target attainment results are summarized in Table 7.

**TABLE 7:** Pharmacokinetic/pharmacodynamic results for vancomycin and meropenem.

PK profile	Trough vancomycin (mg/ml)	AUC/MIC vancomycin	Achieved PK/PD target	Cp t50 meropenem	Achieved PK/PD target
1	17.8	558.3	Yes	20.56	Yes
2	12.7	306.8	No	35.00	Yes
3	18.0	543.0	Yes	64.50	Yes
4	16.1	492.8	Yes	40.90	Yes
5	10.7	239.1	No	18.70	Yes
6	13.3	339.6	No	101.00	Yes
7	16.4	547.4	Yes	73.00	Yes
8	15.3	419.7	Yes	11.20	Yes
9	23.6	599.2	Yes	28.40	Yes
10	14.0	394.0	No	43.20	Yes
11	15.9	440.9	Yes	7.30	No

Area under curve/minimum inhibitory concentration (AUC/MIC); pharmacokinetic/pharmacodynamic (PK/PD); plasma concentration at half a dosing interval (Cp t50).

## Discussion

To the best of our knowledge, this study is the first to perform pharmacokinetic modelling of meropenem concentrations for paediatric patients receiving ECMO. This study also builds on the current, yet limited knowledge, of vancomycin pharmacokinetics in paediatric patients receiving ECMO.

The main findings of this study are that the meropenem clearance was lower than most reported studies, range (0.13–1.053 L.h^−1^.kg^−1^). There is little information available describing the Vd of meropenem in pediatric critically ill patients on ECMO, with meropenem PK data limited to case reports (Cies et al., 2014; Cies et al., 2016; Saito et al., 2020) and one prospective study with larger Vd than our results (Wang et al., 2021). Most of them administered the antibiotic by continuous or prolonged infusion and higher than conventional dosing were used to achieve target PK/PD or a successfull clinical outcome.

Previous studies about PK of meropenem in pediatric critically ill patients without ECMO support shows great dispersion of results (Kongthavonsakul et al., 2016; Cies et al., 2017; Rapp et al., 2020); scientific evidence to establish the influence of ECMO on meropenem PK in this group of patients is missing.

Several publications about pharmacokinetics of vancomycin and pediatric patients on ECMO support, with different designs and conclusions are available. In a retrospective study, Lonabaugh et al. proposed lower dosing than our results, 30 mg/kg/day every 12 h for patients with normal renal function (Lonabaugh et al., 2017). We found similar results for both PK parameters than a retrospective study by Moffett et al., nevertheless **we better** characterized the drug distribution phase, but they proposed lower doses than ours (Moffett et al., 2018).

In a population pharmacokinetic study, Mulla et al. found PK parameters similar than ours and they recommended vancomycin dosing between 15 mg/kg every 8 h and 20 mg/kg every 6 h. However, they included only seven pediatric prospective patients (Mulla and Pooboni, 2005).

Our previous retrospective pharmacokinetic study of vancomycin in pediatric patients on ECMO, reported similar PK parameters to the current study (Zylbersztajn et al., 2018). All studies, including this one, identify a relationship between vancomycin clearance and serum creatinine concentrations and this result is expected as vancomycin is nearly completely renally eliminated (Rybak, 2006).

In our actual study, four PK profile of vancomycin achieved lower PK/PD target, three of them corresponded to a dosing of 15 mg/kg every 6 h. Besides, there was a relationship between trough plasma concentration lower than 15 mcg/mL and AUC/MIC lower than 400. Although no dose modifications were made for the present study, it would be appropriate to consider higher doses in specific patients. PK/PD target of AUC/MIC greater than 400 is the preferred target for treatment of *Staphylococcus aureus* infections in the adult population (Liu et al., 2011), the extrapolation to pediatric population is not clear yet (Le et al., 2013), then, we continue recommending therapeutic drug monitoring for the individualization of dosing in critically ill patients on ECMO.

No other studies have reported population PK data from South American paediatric patients.

Limitations of this study include that a subgroup analysis was not performed to assess the impact of continuous renal replacement therapy (CRRT) on meropenem or vancomycin PK, due to a low number of patients under this technique. Furthermore, in this study there was a limited number of patients or PK profiles, however, we reached a sample size of connections to achieve adequate results (U.S. Department of Health and Human Services, 2019). Another limitation was the lack of clinical outcome evaluation due to the study.

This research contributes to increase the knowledge of the impact of ECMO on pharmacokinetics of vancomycin and meropenem in pediatric patients.

Based on previous studies and the results of this prospective pharmacokinetic study, maximal dosing of meropenem using an extended infusion and at least current dosing of vancomycin with therapeutic drug monitoring are necessary to achieve adequated PK/PD targets in this patient cohort. Nevertheless, larger and prospective studies are needed for robust dosing recommendations.

## Data Availability

The raw data supporting the conclusions of this article will be made available by the authors, without undue reservation.
